# Sexual experience and HIV-related knowledge among Belgian university students: a questionnaire study

**DOI:** 10.1186/1756-0500-7-299

**Published:** 2014-05-15

**Authors:** Sophie Degroote, Dirk Vogelaers, Griet Liefhooghe, Peter Vermeir, Dominique M Vandijck

**Affiliations:** 1 Department of General Internal Medicine, Infectious Diseases and Psychosomatics, Ghent University Hospital, De Pintelaan 185, 9000 Ghent, Belgium; 2 Department of Public Health, Ghent University, De Pintelaan 185, 9000 Ghent, Belgium; 3 Department of Internal Medicine, Ghent University, De Pintelaan 185, 9000 Ghent, Belgium; 4 Faculty of Medicine and Health Sciences, Ghent University, De Pintelaan 185, 9000 Ghent, Belgium; 5 Department of Economics, Hasselt University, Agoralaan Building D, 3590 Diepenbeek, Belgium

**Keywords:** HIV, Acquired Immunodeficiency Syndrome, Knowledge, Sexual behaviour, Adolescent

## Abstract

**Background:**

Adolescents are a risk group for acquiring sexually transmitted diseases, including HIV. Correct knowledge about transmission mechanisms is a prerequisite to taking appropriate precautions to avoid infection. This study aimed at assessing the level of HIV-related knowledge among university students as a first step in developing targeted interventions. We used a self-developed HIV knowledge questionnaire, supplemented with socio-demographic and sexual behaviour questions. The questionnaire was composed of 59 items from different existing questionnaires. It included general statements and statements about prevention, transmission and treatment of HIV.

**Results:**

There were 357 (79.7%) female and 93 (20.3%) male participants and their median age was 20 (IQR 19–21). On average 42/59 (71.2%) questions were answered correctly, 5/59 (8.5%) were answered incorrectly and 12/59 (20.3%) were unknown . The best and worse scores were seen on the prevention questions and the treatment questions, respectively. HIV-related knowledge is higher in older students and in students with a health-related education. Students with sexual experience, with five or more partners and students who have been tested on STDs have a higher HIV-related knowledge.

**Conclusions:**

Knowledge on prevention and transmission of HIV is fairly good among university students and knowledge is higher among students with more sexual experience. They still have some misconceptions (e.g. HIV is spread by mosquitoes) and they are ignorant of a substantial number of statements (e.g. risk for infection through oral sex).

## Background

In Western countries, half of the adolescents is already sexually active at the age of 18 years [1-3]. There is a stabilisation of this number during the last decade, as well as the age at which the first sexual intercourse takes place [1,3,4]. Adolescents are a particular risk group for acquiring sexually transmitted diseases (STDs). Their sex life gets a boost during college days (they gain freedom, go out, meet new people…) while low rates of condom use [5] and experimenting with alcohol and drugs [6] pave the way for unprotected sex. Young adults have a low health risk assessment: they do recognise peers as being at risk for STDs such as HIV, but not themselves [1,7]. This pattern especially emerges in countries with a low prevalence of AIDS [8]. Moreover, HIV infection has become a chronic condition and previous research showed that health-related quality of life in people living with HIV in Belgium is comparable to the general population [9]. Therefore, the fear of getting infected may become less distinct [10].

Consequently, the highest prevalence of STDs in Belgium is seen among 20 to 24-year-old people [5]. Among the 1.100 to 1.200 people in Belgium infected by the HIV virus every year, about 10% is between 15 and 24 years [11]. Specific education and prevention campaigns are still warranted to reduce STD/HIV transmission in this population.

A first, although unsatisfactory, condition to achieve this reduction, is a good knowledge about the transmission of STDs, and HIV in particular. Previous studies have shown gaps in the HIV knowledge of young adults [12-17]. Different interventions to improve this knowledge have been described, with varying degrees of success [18-21]. Improving knowledge can have different secondary goals: reducing sexual risk behaviour, improving condom use or establishing better attitudes towards people living with HIV (PLWH) [22-24].

In Belgium, an HIV knowledge survey was carried out among the general population in 2008 [25]. This was a part of a periodic general health survey with 11.250 participants. The survey included five questions concerning transmission, prevention, testing and attitude towards PLWH. The purpose of this periodic survey is to monitor and to follow the knowledge about HIV in order to observe effects of prevention programs. The survey of 2008 revealed that in the age category of 15–24 years the HIV-related knowledge was lower than in the older group (25 to 54 years). However, this survey is very limited and does not register sexual behaviour as a possible covariate.

More recently, Van Rossem et al. [10] compared AIDS knowledge and sexual activity among secondary school students from different education types in the Northern part of Belgium (Flandres). Students from general education had better AIDS knowledge and were less sexually active than students from vocational education. Within all different types of education, sexually active students had a better AIDS knowledge.

The aim of this study was to assess HIV-related knowledge and sexual behaviour in Belgian university students. Following research questions were addressed: 1) Are there gaps in HIV-related knowledge among this high-educated population? 2) Is HIV-related knowledge related to socio-demographics and/or sexual behaviour?

## Methods

### Participants

Participants in the age group between 18 and 25 were recruited during university classes. Professors were asked to provide 10 minutes of their teaching time for this study. A short presentation was given by the researcher to give practical information. This study was approved by the ethics committee of Ghent University Hospital, as a part of a larger research project, called the HIV CARE Study (Belgian registration number: B670201112850).

### Instrument

The instrument was composed by items from four previously validated questionnaires. 37/45 items from the HIV Knowledge Questionnaire [26] were included in our survey. Eight were not retained because they were not relevant for our Belgian population (e.g. lambskin condoms) or because of the overlap with the transmission questions. Four questions from the AIDS Risk Behaviour Knowledge test [27] were also included. Two questions from the study of Nachega et al. [28] and one item from the HIV Knowledge Scale for Hispanics [29] were ultimately added. The latter examines knowledge about which bodily fluids can transmit the HIV virus. The format of the questionnaire, including the ‘I don’t know’ option, was adopted from previous work in our research group [30-32]. The questions were divided into four categories: general (12 items), transmission (27 items), prevention (12 items) and treatment (8 items).

### Statistical analyses

Data were analysed by means of the statistical package SPSS, version 21 (SPSS, Inc., Chicago IL, US). Categorical data are reported as numbers and percentages and continuous data by medians and interquartile ranges (IQR) (as non-normally distributed). Total scores on the questionnaire and on the four subcategories were calculated. A descriptive analyse of the students’ responses on each of the 59 questions was done. Differences in terms of total score between groups were identified by means of Mann–Whitney-U or Kruskal Wallis tests, if appropriate followed by post-hoc tests.

## Results

### Participant’s characteristics

A total of 450 participants completed the questionnaire, among whom 357 (79.3%) females and 93 (20.7%) males. Median age was 20 (IQR 19–21). Participants’ characteristics are shown in Table 1.

**Table 1:** Characteristics of the participants

	**n**	**%**
**Sex**		
Female	357	79.3
Male	93	20.7
**Age**		
18	104	23.1
19	113	25.1
20	41	9.1
21	104	23.1
22	40	8.9
23	23	5.1
24	17	3.8
25	8	1.8
**Nationality (n = 448)**		
Belgian	442	98.7
Other	6	1.3
**Study (n = 448)**		
Educational sciences	135	30.1
Linguistics and literature	98	21.9
Pharmaceutical sciences	93	20.8
Health care management and policy	43	9.6
Philosophy	26	5.8
Communication sciences	21	4.7
Moral sciences	13	2.9
Other	19	4.2
**Religion**		
Catholic	270	60.1
Jewish	1	0.2
Muslim	3	0.7
Buddhist	2	0.4
No religion	168	37.4
Other	5	1.1
**Sexual orientation (n = 448)**		
Heterosexual	428	95.5
Homosexual	12	2.7
Bisexual	8	1.8
**Relationship**		
Yes	252	56.1
No	197	43.9
**Sexual experience**		
Yes	363	80.8
No	86	19.1
**Number of sex partners**		
0	86	19.2
1	145	32.4
2	83	11.4
3	51	11.4
4	30	6.7
5	19	4.2
> 5	34	7.6
**Contraception women (*n*** **= 292)**		
Contraception pill	246	84.2
Coïtus interruptus	9	3.1
Condom	135	46.2
Spiral	4	1.4
Other	16	5.5
**Contraception men (*n*** **= 70)**		
Contraception pill (female partner)	28	40.0
Coïtus interruptus	1	1.4
Condom	49	70.0
Spiral (female partner)	2	2.9
Other	1	1.4
**STD**		
Yes	6	1.3
No	441	98.7
**Tested on STD’s**		
Yes	76	16.9
No	373	83.1

### Questionnaire scores

The median total score on the 59 items was 42 (37–46), this corresponds with a percentage of 71.2% (62.7%-78.0%). Concerning the subscales, the best score was seen on the prevention items (median 10/12). Transmission and general knowledge scores were slightly lower (21/27 and 9/12 respectively) and the score on the treatment questions was the lowest (3/8) (Table 2).

**Table 2:** Scores on the questionnaire

	**Median**	**IQR**	**%**	**Spread of %**
Total score *(59)*	42	37 – 46	71.2	62.7 – 78.0
General *(12)*	9	8 – 10	75.0	73.3 – 83.3
Transmission *(27)*	21	18 – 23	77.8	66.7 – 85.2
Prevention *(12)*	10	9 – 11	83.3	75.0 – 91.7
Treatment *(8)*	3	2 – 4	37.5	25.0 – 50.0

Number of items is reported in italic, % and spread of % are based on the median and the IQR.

The number and percentage of correct, incorrect and ‘I don’t know’ answers on each statement is shown in an additional file (Additional file 1). Only the most remarkable results will be reported here. Testing seems unfamiliar to many students: a majority (292 students, 65.2%) does not know whether HIV is routinely tested during pap smears, 216 students (48.4%) do not know that HIV infection can not be detected one week after the risk contact and 91 students (20.3%) think that a test lab has to inform the partners of a HIV-positive person.

Some misconceptions about transmission exist as well: 168 (37.2%) participants think that HIV can be spread by mosquitos and 114 (25.7%) do not know. A minority of 203 (45.5%) students are aware of the HIV-risk when having oral sex with a HIV-positive woman. Finally, 170 (37.9%) students incorrectly think that there is a risk for acquiring HIV by donating blood and only 127 (28.7%) of them know that HIV can be transmitted through breast feeding.

The statements about HIV treatment were rarely answered correctly; mostly ‘I don’t know’ was indicated. The preventive value of ART after rape and the effect of missing doses on the transmission risk of HIV were the questions with the fewest correct answers (29, 6.5% and 31, 6.7% respectively).

### Factors associated with knowledge

There is a significant positive correlation between total score and age (Spearman’s rho 0.276, p < 0.01). There are differences according to the education. Students of Health care management and policy (median score 46, IQR 42–50) and Pharmaceutical sciences (median score 45, IQR 42–48) have significantly higher scores than the others (all p < 0.05) and students of Communication sciences (median score 37, IQR 32–39) have significantly lower scores than the others (all p < 0.049).

Students who are currently in a relationship, have significant higher scores than single students (43, IQR 38–46 versus 41, IQR 36–45, p = 0.027). Differences in sexual behaviour are associated with differences in the questionnaire total score as well. Hence, students with sexual experience have higher scores than students without sexual experience (median score 43, IQR 38–46 versus median score 40, IQR 36–45, p = 0.007)*.* Within the group with sexual experience, higher scores are seen in students who have been tested on STDs than those who have not (median score 46, IQR 41–50 versus median score 42, IQR 37–45, p < 0.001). Differences according to the acquisition of STDs were not found (p = 0.401), but this could be due to a limited number of participants who acquired STDs.

The number of sexual partners is also associated with the questionnaire total score. Students reporting five of more partners (median score 44, IQR 41–48) have higher scores than students with two, three or four partners (median score 43, IQR 38–46, p = 0.018) and than those with one partner (median score 42, IQR 37–46, p = 0.003). Religion (p = 0.398), sexual orientation (p = 0.727) and contraception method (all p > 0.05) were not associated with the total questionnaire score.

## Discussion

Although in general, HIV-related knowledge is fairly good in this sample of university students, there are several misconceptions. Myths about transmission via insect bites and donating blood seem to be persistent and have also been found in studies from other countries [8,13,33,34] as well as in the Flemish study [10]. Somewhat contrary to other countries, many Belgian university students do not know that the virus can be transmitted via mother milk (e.g. Chinese students: 78.8% correct answers versus 28.7% in this sample [14]). Knowledge about HIV treatment is poor, probably owing to unfamiliarity with this subject. The low score on this category of questions can be justified, and this knowledge could be considered less essential. Yet, the possibility of post-exposure prophylaxis should be more announced among this risk group.

In accordance with previous research, we found that older students have better HIV-related knowledge [10,14,34,35]. This is possibly due to personal experiences which lead to more interest in this topic [10]. This may also indicate that sexual education in secondary schools is insufficient [35]. More probably, however, students gradually gather information through various channels: television, magazines, peers [14].

Subsequently, the better HIV-related knowledge in sexually active students can be explained by personal involvement. This may lead to active information seeking, or becoming more receptive to information [10]. It is possible that students who have more lifetime sexual partners, are more aware of the risk for STDs. This assumption is supported by additional analyses in this sample. The relative number of students using condoms as well as the number of students who already had a STD-test, was linearly associated with the number of partners.

Engaging in sexual risk behaviour is not always associated with inadequate knowledge and vice versa [8]. Therefore, it seems that providing information alone is not effective to reduce sexual risk behaviour [15,18,36]. Nevertheless, improved knowledge can make students feel more comfortable to talk about the topic in a relationship [37]. In their review, Scott-Sheldon et al. concluded that behavioural interventions can be efficient to reduce STDs and to improve condom use. Besides education, behavioural interventions include motivational training and/or skills training (e.g. partner negotiating, condom practice on a model) [38]. Almost two decades earlier, similar strategies were suggested for school-based programs in a review of Kirby et al. [39]. Moreover, the programs should have a focus on specific behavioural goals, use social learning (e.g. modelling) and address social and media influences on sexual behaviour [39]. A last positive feature of intervention programs could be peer-learning. An instructor of the same age category can facilitate talking and discussing about sexual behaviour and can serve as a role model [22]. For adolescents, peers are a main source of information about such topics [15].

We found no association between religion and HIV-related knowledge. In Belgium, this is not surprising since few people practice their religion [40]. It is unclear whether students reporting to be catholic have other values and ideas about (sexual) relationships than students reporting to be non-religious.

This study has certain limitations. First of all, our sample of university students is not representative for all university students in Flanders (Belgium) (e.g. only 20.7% males versus ±40% [41]) and did not include all types of education. More specifically, no students from the faculties of sciences, engineering and economics were included. Future research could also include adolescents following non-university higher education and/or adolescents not following higher education to explore possible knowledge differences according to educational level. Secondly, the self-developed knowledge instrument was not validated, but this was considered legitimate as it was composed by statements of previously validated questionnaires. Because of the detailed and extensive knowledge instrument, sexual risk behaviour was assessed only through indicative questions such as the number of partners and contraception and not through an additional questionnaire. Lastly, the cross-sectional nature of the study does not reveal causal associations or predictors of good HIV-related knowledge.

Strengths of this study include the detailed information resulting from the rather extensive questionnaire. To avoid guessing, students could indicate the option ‘I don’t know’. This distinguishes the non-correct answers into misconceptions and doubts. This is interesting since these could be addressed differently in campaigns or interventions.

## Conclusions

Knowledge on prevention and transmission of HIV is fairly good among Belgian university students and is comparable to those found in other countries. There is, however, room for improvement. Older and sexually experienced students have a better knowledge, most likely due to more personal involvement. Future interventions should approach both sexually experienced and non-experienced students with tailored information and skills.

## Competing interests

The authors declare that they have no competing interests.

## Authors’ contributions

SD participated in the design and coordination of the study and drafted the manuscript. DV participated in the design of the study and helped to draft the manuscript. GL participated in the design of the study and was responsible for the data collection. PV participated in the coordination of the study. DMV conceived of the study, participated in the design and coordination of the study and helped to draft the manuscript. All authors read and approved the final manuscript.

## Supplementary Material

Additional file 1 This table shows all statements of the questionnaire (including source) and the number and % of students who answered correctly, incorrectly or ‘I don’t know’. Click here for file
